# Effects of Clockwise and Counterclockwise Job Shift Work Rotation on Sleep and Work-Life Balance on Hospital Nurses

**DOI:** 10.3390/ijerph15092038

**Published:** 2018-09-18

**Authors:** Dana Shiffer, Maura Minonzio, Franca Dipaola, Mattia Bertola, Antonio Roberto Zamuner, Laura Adelaide Dalla Vecchia, Monica Solbiati, Giorgio Costantino, Raffaello Furlan, Franca Barbic

**Affiliations:** 1 Internal Medicine Humanitas Clinical and Research Center, 20089 Rozzano, Italy; danaalon@yahoo.com (D.S.); maura.minonzio@humanitas.it (M.M.); franca.dipaola@humanitas.it (F.D.); raffaello.furlan@hunimed.eu (R.F.); 2 Biomedical Sciences Department, Humanitas University, 20089 Rozzano, Italy; 3 Surgery Department, Borgomanero Hospital, ASL Novara, 28021 Borgomanero, Italy; mattia.bertola@alice.it; 4 Departamento de Kinesiologia, Universidad Catolica del Maule, Talca 3480112, Chile; beto.zam@gmail.com; 5 IRCCS Istituti Clinici Scientifici Maugeri, 20138 Milan, Italy; laura.dallavecchia@icsmaugeri.it; 6 Fondazione IRCCS Ca’ Granda Ospedale Maggiore Policlinico, University of Milan, 20122 Milan, Italy; monica.solbiati@gmail.com (M.S.); giorgic2@gmail.com (G.C.)

**Keywords:** shift work, sleep disturbances, work-life balance, hospital nurses, clockwise and counterclockwise shift rotation

## Abstract

Rotational shift work is associated with sleep disturbances, increased risk of cardiovascular and psychological disorders, and may negatively impact work–life balance. The direction of shift rotation (Clockwise, CW or counterclockwise, CCW) and its role in these disorders are poorly understood. The aim of the study was to investigate the effect of the shift schedule direction on sleep quantity and quality, alertness and work performance, and on work–life balance on hospital nurses. One-hundred female nurses, working a continuous rapid shift schedule in hospitals in the north of Italy, participated in this cross-sectional study. Fifty worked on CW rotation schedule (Morning: 6 a.m.–2 p.m., Afternoon: 2 p.m.–10 p.m., Night: 10 p.m.–6 a.m., 2 rest days) and fifty on CCW rotation (Afternoon, Morning, Morning, Night, 3 rest days). Data were collected by ad hoc questionnaire and daily diary. During the shift cycle CW nurses slept longer (7.40 ± 2.24 h) than CCW (6.09 ± 1.73; *p* < 0.001). CW nurses reported less frequently than CCW awakening during sleep (40% vs. 80%; *p* < 0.001), attention disturbance during work (20% vs. 64%; *p* < 0.001), and interference with social and family life (60% vs. 96% and 20% vs. 70%, respectively; *p* < 0.001). CCW rotating shift schedule seems to be characterized by higher sleep disturbances and a worse work–life balance.

## 1. Introduction

Working on a shift system, i.e., outside the regular 9 a.m. to 5 p.m. working hours, is frequently required in work places which operate on a 24-h schedule [1]. A wide variety of shift work schedules currently exist to accommodate the needs and expectations of modern society. Schedules may differ by length, number of consecutive shifts or shifts per week, speed, and/or direction of rotation and the presence or absence of night work [1]. 

The adverse effects of shift work on the workers’ health and well-being has long been a subject of several previous studies [2,3,4,5,6,7,8]. Rotational shift work is associated with an increased risk of metabolic disorders, cardiovascular abnormalities, cancer, and psychological disorders [2,3,4,5,6,7,8]. In addition, disturbances in sleep and alertness are frequently reported by individuals who work shifts and, in particular, by those working night shifts [9,10]. These alterations may be explained by a misalignment between the physical and psychological requirements of the shift work schedule and the oscillations of biochemical, physiological, and behavioral variables which are controlled by the endogenous circadian clock [11,12]. Day–night oscillations of body temperature, hormone secretions, blood pressure, and sleep–wake rhythm may all become altered by shift work [12,13,14]. This may result in less sleep time and poor sleep quality [15,16,17]. Insufficient sleep and consequent fatigue may impair cognitive function and increase risk of work-related errors and accidents [18,19]. Shift work has also been shown to have a negative impact on family and social life [20,21] since working at irregular hours may limit the available time that can be spent with family and friends, ultimately leading to work–family/social conflict. To minimize the above-mentioned adverse effects the search for a better shift work design is ongoing. However, consensus on the matter lacks as previous studies have produced conflicting results [22,23,24,25,26,27,28,29,30]. 

Hospitals commonly require the health care workers to work on a shift schedule to ensure continuity of care. Nurses make up an essential part of the health care team and have a wide range of responsibilities which involve constant monitoring and care of patients. They not only face the challenging nature of the job itself but also the hardships and the various effects associated with shift work. It is common for hospital nurses to work on rapidly rotating shift systems which may vary by direction of rotation. They may follow a clockwise (CW) (forwards) rotation direction (i.e., day, afternoon, night) or a counterclockwise (CCW) (backwards) rotation direction (i.e., day, night, afternoon). The effect on the workers’ physical and mental health due to the different direction of rotation is inconclusive. Several studies have shown that a CCW rotation schedule was associated with poorer sleep quality and quantity [31,32,33,34]. Other studies, however, have failed to show that the direction of shift rotation had differing repercussions on sleep, work performance, and on work–life balance [24,35,36]. 

The aim of the present study was to investigate if the direction of rapid shift rotation schedules affects hospital nurses’ sleep quantity and quality, work performance, and its impact on their social and family life. The understanding of these aspects is crucial for improving work schedule organization of hospital nurses in order to promote health and wellness and reduce the shift work maladaptation.

## 2. Methods

### 2.1. Study Population

**Figure 1:**
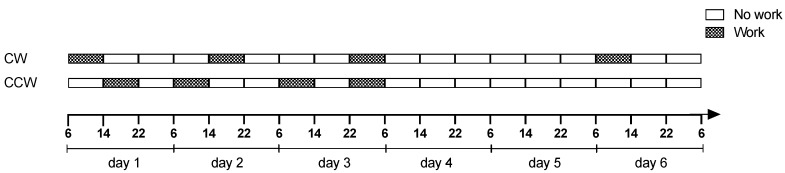
Work schedule of CW (Clockwise) and CCW (counterclockwise) rotating nurses over an entire shift cycle.

This cross-sectional study was conducted in two major hospitals in Northern Italy with the voluntary participation of 100 registered female nurses, 50 from each hospital corresponding to approximately 80% of the total eligible workforce. As shown in Figure 1, the group of 50 nurses in Hospital 1 had been working on a continuous, rapid CW rotation schedule with the following sequence: Morning (6 a.m.–2 p.m.), Afternoon (2 p.m.–10 p.m.), Night (10 p.m.–6 a.m.) followed by 2 rest days (48 h). The other group of 50 nurses, in Hospital 2, had been working on a continuous, rapid CCW rotation schedule with the following sequence. Afternoon (2 p.m.–10 p.m.), Morning (6 a.m.–2 p.m.), Morning (6 a.m.–2 p.m.)/Night (10 p.m.–6 a.m.) followed by 3 rest days (80 h). Both groups worked on an 8-h shift system.

In order to minimize heterogeneity between the two groups, all nurses participating in the study were normotensive and normal-weight (body mass index (BMI) 18–23 kg/m^2^) and they were deemed eligible for shift work by their respective employing hospitals. In addition, the two groups of nurses were homogeneously enrolled from the same following wards of the two hospitals: Internal Medicine (13/50), Nephrology 12/50), Surgery 13/50, and Cardiology (12/50). Thus, it was assumed that nurses’ job tasks were characterized by comparable physical and cognitive requirements. The local organization of nursing staff decided on the CW shift rotation for the nurses working in Hospital 1 and CCW shift rotation for nurses working in Hospital 2.

Ethical approval was granted by the Local Ethics Committees of the target hospitals. All subjects participating in the study provided an informed consent (ethical approval code: SWNURS-633/CE). 

### 2.2. Data Collection

An ad hoc questionnaire and a daily diary were specially designed for the study with the purpose to assess several aspects that would help determine the nurses’ psychological and physical well-being. Specifically, the quantity and quality of sleep, work performance, and attitude, their level of satisfaction with their social/family life and lifestyle habits were evaluated.

A self-administered questionnaire in a closed question format was answered anonymously by each nurse at the onset of the study. The questionnaire was used to obtain general information about number of years in shift work, whether they had children, sleep quantity and quality, work performance, perception of well-being, lifestyle habits, and overall satisfaction with family and social relationships. 

Sleep quantity was calculated as the mean hours during the entire shift cycle. Sleep duration after the night shift was determined by asking about the hours (h) slept on average. (Responses grouped as <6 h, 6–9 h, >9 h).

Sleep quality was evaluated by inquiring about sleep disturbances and by how rested the participant felt. The following questions were asked: ‘Do you have frequent awakening episodes during sleep?’: ‘Do you have difficulty falling asleep?’: ‘Do you feel rested at the start of the shift?’ (yes/no response).

Effects on lifestyle, family, and social life were assessed from questions such as: ‘Do you feel that work interferes with your family life?’: ‘Do you feel that work interferes with your social life?’: ‘Do you find it difficult to manage domestic responsibilities?’ (yes/no response).

Effects on alertness and performance during shifts were evaluated through questions such as: ‘Do you have frequent episodes of inattention at work?’: ‘Do you find it difficult at times to keep up with the work load?’ (yes/no response): ‘During which shift do you have more problems concentrating?’ (morning/afternoon/night response).

In addition, the nurses had to fill out a daily diary at a prefixed time (between 12 noon–3 p.m.) on each work day and on rest days, during one rotation cycle which was 6 days long. Therefore, a total of six daily diaries have been filled out by each nurse during the experimental protocol. The diary, which was kept anonymous, contained questions about the participant’s principle activities in the preceding 24 h. Specifically, they were requested to report the number of hours they had slept in the preceding 24 h and to report the number of hours they slept before and after a night shift, when appropriate. Information about sleep quality was obtained by reporting whether they felt rested at the start of the shift (yes/no). In addition, they were asked about the number of coffee cups consumed in the preceding 24 h and, when relevant, during the night shift. 

Each participant returned the completed questionnaire and diaries in a sealed envelope. Once collected, the data was registered and statistically analyzed with the aim of specifically investigating the effect of the direction of rapid shift rotation. 

### 2.3. Statistical Analysis

Statistical analyses were performed on SPSS 20.0 (SPSS, Inc., Chicago, IL, USA) and GraphPad Prism 7.0 (GraphPad Software, San Diego, CA, USA). Data normality was verified by Kolmogorov–Smirnov test. The data obtained from the questionnaire and diary were analyzed using the Chi-square test for categorical variable. A *t*-student test was applied for between group comparison regarding continuous variables. In order to check whether “age”, “having children”, and “work experience” were associated with the main outcomes in the present study, Phi (for two dichotomous variables) and point-biserial (for one dichotomic and one continuous variable) coefficients were performed. Significance level was set at 5%. 

## 3. Results

The demographic characteristics of the two groups of nurses are reported in Table 1.

The two groups did not differ significantly by age and years of shift work experience. The percentage of nurses who had children was similar in both groups. In addition, there were no significant differences between the groups regarding the percentage of smokers and coffee consumers.

Table 2 and Table 3 display the results and significance levels for each of the variables assessed. 

**Figure 2:**
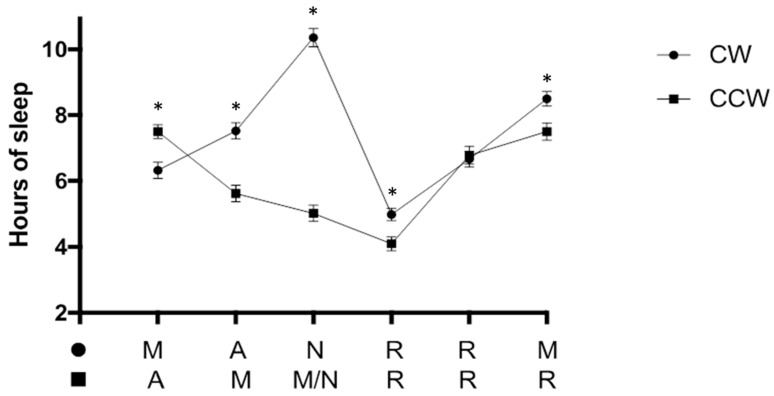
Hours of sleep in the 24 h before each shift or rest days. Note that the CW (clockwise) rotating nurses slept an average of 10 h before the night shift. The CCW (counterclockwise) rotating nurses had a morning shift and a night shift on the 3rd day of the shift cycle and slept an average of 5 h in the 24 h preceding the night shift. Of interest, both the CW and CCW rotating nurses slept the lowest number of hours following the night shift (5 h and 4 h, respectively). M: morning shift (6 a.m.–2 p.m.); A: afternoon shift (2 p.m.–10 p.m.); N: Night shift (10 p.m.–6 a.m.); R: Rest days; M/N: indicates where CCW nurses worked both morning and night shift on the same day; * *p* < 0.05 CW vs. CCW.

Regarding sleep measures, the results show that the nurses working on a CW rotation schedule slept significantly more hours during the period of a shift cycle than those working on a CCW rotation schedule (Table 2). It is worth noting that before the night shift a higher percentage of nurses on a CCW schedule slept less than 6 h (Table 3). Although both groups slept the lowest number of hours after the night shift (Figure 2), the CW rotating nurses still slept significantly more after night shifts compared to CCW rotating nurses (Table 3). Concerning sleep quality, abrupt awakening during sleep was reported to occur more frequently by nurses in the CCW compared to CW rotation (Table 2).

When comparing work performance and alertness between the two groups, a significantly larger number of nurses working on CW rotations reported feeling rested at the beginning of a shift than those working on CCW rotations (Table 2). Whereas, difficulty maintaining attention levels during work hours was perceived to occur more frequently by nurses rotating CCW (Table 2). Specifically, nurses working the CCW schedule reported having greater difficulty maintaining attention levels during the night shift compared to nurses in the CW group (Table 3). CCW rotating nurses consumed significantly more coffee during the night shift than the CW rotating nurses (Table 3).

With regards to work–life balance, a higher number of CCW rotating nurses stated that work had more influence on their private life and was perceived to disrupt family and social relationships (Table 2). There were no significant differences between the two groups regarding managing house related chores and the average number of coffee cups consumed over the entire shift cycle (Table 2). 

Correlation analyses were applied in order to check whether “age”, “shift work experience”, and “having children” were associated with “Sleep < 6 h after night shift”, “Sleep duration before night shift”, “Sleep duration after night shift”, and “Difficulty in concentrating during night shift”. No significant relationships were found (*p* > 0.05).

## 4. Discussion

The results of the present study indicate that working the specific type of rapid CCW rotating schedule has more negative effects on quantity and quality of sleep and work alertness. In addition, CCW rotating schedule impacts more negatively on social and family life than working a rapid CW rotating schedule. Two ad hoc tools (Questionnaire and Daily Diary) were designed by the researchers specifically for the purpose of the present study. This allowed specific data collection in a short and simplified way and facilitated the compliance of the nurses, despite the fact that they have not yet been validated.

### 4.1. Impact on Sleep Duration and Quality

This study shows that nurses working a CCW rotating schedule sleep significantly less hours per day than those working a CW rotation schedule and, also experience significantly more fragmented sleep. This is in line with previous studies [31,37,38]. Indeed, the retrospective study conducted by Shon and colleagues [31] on a group of blue-collar workers to investigate the effects of direction of rotation on sleep quality showed that the CCW schedule was associated with poorer sleep quality. However, the speed of rotation and the intervals of shift rotation were not identified in the study [31]. Similar findings were reported in a survey study by Barton et al. [39] on 261 workers from five different industries. They had found that a CCW system was associated with more sleep disturbances, particularly when the CCW rotation schedule included ‘quick returns’ (8-h break in between shifts). This was also the type of shift schedule the CCW group in the present study worked with, and with similar results. Indeed, in CCW system the break between the afternoon and morning shift and between morning and night shift lasted 8 h (Figure 1). The findings of the present study regarding the nurses’ sleep duration before the night shift are in keeping with those reported by Cruz et al. [36] in their study on air traffic control specialists. That study also showed that workers on a rapid CW rotation schedule slept more before the night shift [36]. 

The greater reduction in the quantity and quality of sleep experienced by the nurses on the CCW rotation schedule could be due to the differences in the intervals between consecutive shift blocks which result from the differing direction of rotation in the two systems. This interpretation would reinforce the suggestion by Barton et al. [39] that the problem may not be due to the direction of shift rotation alone but rather to a combination of rotation direction and lengths of breaks between one shift and the next one [39]. 

In the present study the CW rotation schedule offered longer break intervals (24 h) between consecutive shifts and had a 48-h break interval at the end of the 3-shift blocks. This working schedule is in accordance with the Directive from European Community (Directive 2003/88/EC—working time). In contrast, the CCW rotation schedule had an 8-h break between the afternoon and morning shift, a 16-h break between the morning and the following morning shift and an 8-h break between the morning and night shift and a longer rest period of 80 h at the end of the shift cycle. However, although nurses working on a CCW schedule had a longer rest period at the end of the shift cycle (three days off), which should allow sleep recovery, the ‘quick returns’ from one shift to the next one during work days seem to restrict sleep duration and recovery resulting in fatigue accumulation. In addition, sleep time may be further restricted during those short breaks if there are children to care for at home. Furthermore, the longer break intervals between shifts on the CW rotation schedule may allow for more adequate rest and sleep recovery than the CCW schedule. 

The present study found that, following the night shift, both groups slept the least number of hours even though they were on a rest day. This is likely due to an attempt to sleep during the day when stress hormone levels such as corticosteroids and catecholamines are at the highest levels [23] under the influence of the light–dark cycle and other environmental cues which promote wakefulness [40]. In accordance with this observation, a previous study by Furlan et al. [13] on steel industry shift workers rotating on a CCW direction found that daytime sleep following the night shift was significantly less (5 h) than the hours of sleep following the morning and afternoon shifts (6 and 8 h, respectively). The authors concluded that this observation could be due to a mismatch between circadian rhythms of stress-related biological variables and the work–sleep schedule. 

Previous studies showed that shortened and fragmented sleep can lead to fatigue and decreased cognitive performances [11,41,42]. This might explain why more nurses working a CCW rotation reported feeling less rested at shift onset and had more difficulty maintaining attention levels at work. A significantly higher percentage of nurses from the CCW group reported the most difficulty concentrating during the night shift. This is probably due to having had a break interval of only 8 h after having worked the morning shift. In contrast, nurses on a CW schedule, who had a longer break from the previous shift and slept about 10 h before the night shift, seemed to perform better at work. The CCW nurses of this study, in addition to being out of sync with the day–night cycle, were required to work a CCW schedule which included quick returns. The combination of these factors might further exacerbate cognitive impairment during night shifts [16,43]. Furthermore, the increased coffee consumption during the night shift by CCW rotating nurses suggests its use as a countermeasure to sleepiness. 

Sleep loss has been shown to decrease cognitive capabilities such as concentration, attention span, and reaction time which, in turn, increase the risk of work related errors and accidents [40,44]. This is of great importance since nurses must maintain maximum levels of vigilance and attention during work to ensure efficient patient care and safety. In addition, it is well-known that sleep deprivation is associated with alteration of cardiovascular autonomic control characterized by a chronic sympathetic overactivity that may promote cardiovascular diseases [45].

### 4.2. Impact on Work–Life Balance

The present study indicates that a rapid CCW direction rotation schedule is associated with increased work–family/social conflict. Nurses, working on CCW rotation schedules, were significantly less satisfied with their social and family life. This is in keeping with previous studies [38,39]. A longitudinal prospective study performed by Van Amelsvoort et al. [38] which included 776 three-shift workers (mostly males) concluded that working on a CW rotation led to less work–family conflict than on a CCW rotation. Similarly, a study by Barton et al. [39] reported that workers on a CCW schedule experienced more social and domestic disruption than workers on a CW schedule. Shift work, in general, obliges the worker to work irregular hours leaving a period of free time which is out of sync with the free time of most other people [46]. In addition, if the quick returns associated with the CCW rotation schedule make it harder to sleep sufficiently and have time for family and friends, this working organization may lead to lower work and life satisfaction. Therefore, it seems that a CW rotation schedule allows a healthier balance between work and private life, ultimately leading to a better quality of family and social life.

### 4.3. Strength and Limitations of the Study

Data on the effect of direction of rotation on female subjects is still limited since most of the literature includes studies performed on predominantly male populations. Given that men and women may have different roles in their domestic life, the effects of the direction of rotation could potentially yield different results in different populations. This study, therefore, adds to the limited existing data on the influence of the direction of rotation on sleep measures, work performance, and social and domestic life on an entirely female population. The sample size of this study might represent a potential limitation of the study. On the other hand, the homogenous characteristics of the study population enrolled (sex, age, healthy conditions) minimize the potential confounders [47] and thus represent a strength of the study. However, to ensure appropriate generalizations of the study findings further additional ad hoc surveys might be required. Additionally, as the study was performed on Italian nurses it might not reflect how the direction of shift rotation influences domestic life in other countries and cultures. It is important to mention that since the data was obtained from a self-reporting questionnaire it could be subject to response bias. In the aims of minimizing potential confounding factors, the two groups of nurses in the study worked in comparable medical wards with the same mental and physical work requirements and the diary was required to be filled out by all participants at same hours, every day, over the entire week cycle. Finally, the Questionnaire and Daily Diary designed for this study’s purpose have not yet been validated.

## 5. Conclusions

The results of the study suggest that the direction of a rapid shift rotation schedule in hospital nurses has a significant impact on the sleep quantity and quality as well as on work performance, family and social relationships. Specifically, the impact is greater by a CCW rotation schedule which includes short break intervals between shift blocks. Nurses working on rapid CCW rotating schedules sleep less, suffer more sleep disturbances, and have more troubles in maintaining alertness levels during work hours than nurses working on rapid CW rotation schedules. Furthermore, working on a rapid CCW rotation schedule seems to be associated with greater difficulty in balancing work and social/family life. The results suggest that CW shift rotation may promote a more favorable balance between work activity and private life. Overall, it seems that the negative influences associated with a CCW rotating shift schedule may be due to the combination of the direction of shift rotation and the presence of quick returns.

## Figures and Tables

**Table 1:** Demographics of the study population.

Variables	CW (*n* = 50)	CCW (*n* = 50)	*p*-Value
Age (years)	30 ± 6	30 ± 5	1.00
Shift work experience (years)	9 ± 6	9 ± 5	1.00
Have children, *n* (%)	38 (76)	37 (74)	0.82
Consume coffee, *n* (%)	50 (100)	50 (100)	1.00
Current smokers, *n* (%)	31 (62)	30 (60)	0.84

CW: clockwise; CCW: counterclockwise; Values are expressed as mean ± Standard Deviation and *n* (%) when appropriate.

**Table 2:** Sleep duration, work performance, social and family life characteristics in the clockwise (CW) and counterclockwise (CCW) rotating groups.

Variables	CW (*n* = 50)	CCW (*n* = 50)	*p*-Value
Sleep duration over entire shift cycle (hours)	7.40 ± 2.24	6.09 ± 1.73	<0.0001
Frequent awakening episodes during sleep, *n* (%)	20 (40)	40 (80)	<0.0001
Feel rested at shift start, *n* (%)	35 (70)	8 (16)	<0.0001
Difficulty in concentrating at work, *n* (%)	10 (20)	32 (64)	<0.0001
Work interferes with family life, *n* (%)	30 (60)	48 (96)	<0.0001
Work interferes with social life, *n* (%)	10 (20)	35 (70)	<0.0001
Work interferes with house chores, *n* (%)	35 (70)	36 (72)	0.83
Number of coffee cups consumed over a shift cycle	3.4 ± 1.3	3.7 ± 1.5	0.28

Values are expressed as mean ± Standard Deviation (SD) and *n* (%) when appropriate.

**Table 3:** Sleep features and work performance with respect to the night shift in the CW (clockwise) and CCW (counterclockwise) rotating groups.

Variables	CW (*n* = 50)	CCW (*n* = 50)	*p*-Value
Sleep < 6 h after night shift, *n* (%)	10 (20)	28 (56)	0.0004
Sleep duration before night shift (h)	10.36 ± 1.7	5.02 ± 1.2	<0.0001
Sleep duration after night shift (h)	4.98 ± 1.5	4.1 ± 1.3	0.0022
Difficulty in concentrating during night shift, *n* (%)	10 (20)	36 (72)	<0.0001
Coffee cups consumed during night shift	3.14 ± 1.3	4.36 ± 1.4	<0.0001

Values are expressed as *n* (%) and mean ± SD when appropriate.
